# Real-World Treatment Patterns and Clinical Outcomes in Canadian Patients with AML Unfit for First-Line Intensive Chemotherapy

**DOI:** 10.3390/curroncol29100535

**Published:** 2022-09-22

**Authors:** David Sanford, Pierre Desjardins, Brian Leber, Kristjan Paulson, Sarit Assouline, Paola M. C. Lembo, Pierre-André Fournier, Heather A. Leitch

**Affiliations:** 1 Vancouver General Hospital, University of British Columbia, Vancouver, BC V5Z 1M9, Canada; 2 Hémato-Oncologue, Centre Intégré de Cancérologie de la Montérégie, Hôpital Charles LeMoyne, Greenfield Park, QC J4V 2G9, Canada; 3 McMaster University Medical Centre, McMaster University, Hamilton, ON L8S 4L7, Canada; 4 Max Rady College of Medicine, University of Manitoba, Winnipeg, MB R3A 1R9, Canada; 5 Jewish General Hospital, McGill University, Montreal, QC H3T 1E2, Canada; 6 AbbVie Corporation, Saint-Laurent, QC H4S 1Z1, Canada; 7 St. Paul’s Hospital, University of British Columbia, Vancouver, BC V6E 1M7, Canada

**Keywords:** acute myeloid leukemia, real-world evidence, treatment patterns, chemotherapy-ineligible, outcomes

## Abstract

Acute myeloid leukemia (AML) is a hematological malignancy that predominantly affects the elderly. Prognosis declines with age. For those who cannot tolerate intensive chemotherapy, historically established treatment options have been hypomethylating agents (HMAs), low dose cytarabine (LDAC), and best supportive care (BSC). As the standard of care evolves for those unfit for intensive chemotherapy, there is a need to understand established treatment pathways, clinical outcomes and healthcare resource utilization in Canada. The CURRENT study was a retrospective chart review of AML patients not eligible for intensive chemotherapy who initiated first-line treatment between 1 January 2015 and 31 December 2018. Data were collected from 170 Canadian patients treated at six hematology centers, of whom 118 received systemic therapy and 52 received BSC as first-line treatment. Median overall survival was 8.58 months and varied from 2.96 months for BSC to 13.31 months for HMAs. Over 80% of patients had at least one outpatient visit, and 67% of patients receiving systemic therapy and 71% of those receiving BSC had at least one admission to hospital, during their first line of therapy. A total of 96 (81.4%) patients receiving first line systemic therapy and 39 (75.0%) of those receiving first line BSC had at least one red blood cell or platelet transfusion. These findings highlight the unmet need for novel therapies for patients ineligible for intensive chemotherapy.

## 1. Introduction

Acute myeloid leukemia (AML) is a hematologic malignancy characterized by rapid proliferation of undifferentiated myeloid cells in the blood, bone marrow and other tissues [1]. The median age at AML diagnosis is 68 years [2]. Overall, 5-year survival rates have been reported to range from 19–29%. Nevertheless, in clinical practice, it is necessary to consider patient preferences in decision making and occasionally patients will elect for BSC over other treatment options [2,3,4]. A recent US-based population study estimated 5-year survival rates to be 63% for patients aged 15 to 39 years, but declining to 22% in those aged 60 to 69 years and to 5% amongst those aged over 70 years at the time of diagnosis [5]. Induction chemotherapy followed by post-remission (consolidation) therapy or allogeneic stem cell transplant (SCT) is the standard of care for those who can tolerate such intensive treatment, resulting in three-year overall survival (OS) rates of 54–58% [6,7,8]. Historically, median OS in older AML patients has been poor and a previous registry based study reported OS of 184 days for patients aged 66–75 and 80 days for those aged over 76 years [9]. This is in part due to of the age-related increase in frequency of AML with adverse-risk genetics and secondary AML, and/or multidrug resistance, and an inability to physically tolerate intensive chemotherapy due to comorbidities or frailty (the latter serving to highlight the need for less aggressive yet effective treatment regimens to extend benefit to those currently considered unsuitable for existing options) [6,10,11].

Historically, for patients who are ineligible for intensive chemotherapy the median estimated survival is less than one year [6,7,12]. Standard treatment options for these patients have been the hypomethylating agents (HMA) 5-azacitidine (azacitidine)/decitabine, or low-dose cytarabine (LDAC) and best supportive care (BSC) with hydroxyurea or transfusion support [1]. Recently, several novel therapies have been introduced as alternatives for AML patients ineligible for intensive chemotherapy. These include venetoclax, a BCL-2 inhibitor, and glasdegib, a hedgehog pathway inhibitor, both approved by the European Medicines Agency, US Food and Drug Administration and Health Canada (amongst others). Venetoclax is for use with a hypomethylating agent or with LDAC for treatment-naïve elderly AML patients who are ineligible for intensive chemotherapy; glasdegib is approved in combination with low-dose cytarabine, for the treatment of newly diagnosed AML in patients ≥75 years old or who have comorbidities that preclude use of intensive induction chemotherapy [13,14,15,16,17,18].

As the standard of care (SOC) evolves with novel therapies and rising costs of treatment, there is a need to understand current AML treatment pathways, clinical outcomes including survival, clinicopathologic characteristics, and healthcare resource utilization (HRU) of patients unfit to receive intensive chemotherapy in clinical practice.

## 2. Materials and Methods

The Real-World Treatment Patterns and Clinical Outcomes in Unfit AML Patients Receiving First Line Systemic Treatment or Best Supportive Care (CURRENT) study was a multicenter, multinational non-interventional retrospective chart review designed to understand the clinicopathologic characteristics, treatment patterns, clinical outcomes (including survival), and HRU of AML patients who are unfit to receive intensive chemotherapy in real-world clinical practice. Overall results of the global study have been published elsewhere [19,20]. The focus of this manuscript is the results for the Canadian dataset from the global study.

The study was conducted in accordance with the principles of Good Pharmacoepidemiology Practices and the ethical principles that have their origin in the Declaration of Helsinki. Institutional Review Board/Ethics Committee (IRB/EC) approval was obtained prior to the initiation of the study as necessary per local regulations.

Anonymized medical records of Canadian adults (>18 years old) diagnosed with primary or secondary AML between January 01, 2015 and December 31, 2018 were eligible for data extraction if they were deemed ineligible for intensive chemotherapy (Patients could be defined as ineligible for intensive induction therapy on the basis of their treating physician’s assessment of age, Eastern Cooperative Oncology Group (ECOG) performance status, comorbidities, regional guidelines, institutional practice, or all of the above [21]), had received first-line systemic therapy (including low intensity chemotherapy), targeted therapy or BSC, and had at least two physician visits after starting therapy. Those for whom a diagnosis of AML was not confirmed, or had acute promyelocytic leukemia, and who received first line therapy as part of a clinical trial, were excluded. Data were entered into an online system and a secure database for analysis, storage and reporting.

The primary endpoint for the study was OS. Secondary endpoints were progression free survival (PFS), time to treatment failure (TTF), HRU, measurable residual disease (MRD) testing rates including methodology as available, and rates of complete remission (CR), time to achieve CR, duration of CR, CR with incomplete hematologic recovery (CRi), morphologic leukemia free state (MLFS), partial remission (PR), and treatment failure [7].

The CURRENT study aimed to capture data from approximately 1600 patients being treated at 175 sites in 30 countries. As the study was descriptive in nature, no formal hypothesis testing or power calculations were required. Data were summarized using descriptive statistics and the Kaplan–Meier method was used to estimate proportions and median times for time-to-event analyses (OS, PFS, TTF). Kaplan–Meier curves were presented with two-sided 95% confidence intervals. Differences between subgroups (treatment, risk factors, geography) were explored with log-rank tests and Cox regressions. To mitigate possible sampling bias during site and patient recruitment, specialist sites across Canada were approached to participate in the study. For sites that identified more eligible patients than their enrolment target, instructions for a random sampling method were provided.

## 3. Results

### 3.1. Study Population

In the overall CURRENT study, data were collected for 1762 patients with AML from 22 countries. In Canada, data were collected from 170 patients treated at six hematology centers, of whom 118 (69.4%) received systemic therapy and 52 (30.6%) received BSC as first-line treatment. These proportions are broadly similar to those for the global CURRENT cohort (74% and 26%, respectively). Demographics and baseline characteristics were similar for the two treatment groups (Table 1). Ethnicity was not included as it is not typically captured in patient records in Canada. Overall, 41.8% were aged >75 years, 42.9% had secondary AML, 63.5% had intermediate or poor risk cytogenetics. Of the 96 patients with molecular data, 40 had ≥1 mutation, the most common of which were NPM1 (13 patients) and JAK2 (7 patients). Of those with ECOG performance status available, the proportion with ECOG ≥2 was higher for those receiving BSC (62.2%) vs. those receiving systemic therapy (42.6%), although there were more patients in the systemic therapy group with missing data (60.2% vs. 28.8% for BSC). Approximately 10% were hospitalized for leukemia treatment initiation. In the global CURRENT cohort the proportion of patients aged 75 years and older who received BSC was 61% (compared with 53.8% in the Canadian dataset). Fewer patients in the global cohort had unknown ECOG status (10–22% across treatment groups) compared with the Canadian dataset (28.8–60.2%) while more patients had an unknown cytogenetic risk (23–53% in the Global cohort vs. 12.7–40.4% in the Canadian dataset).

### 3.2. Treatment Patterns

**Figure 1:**
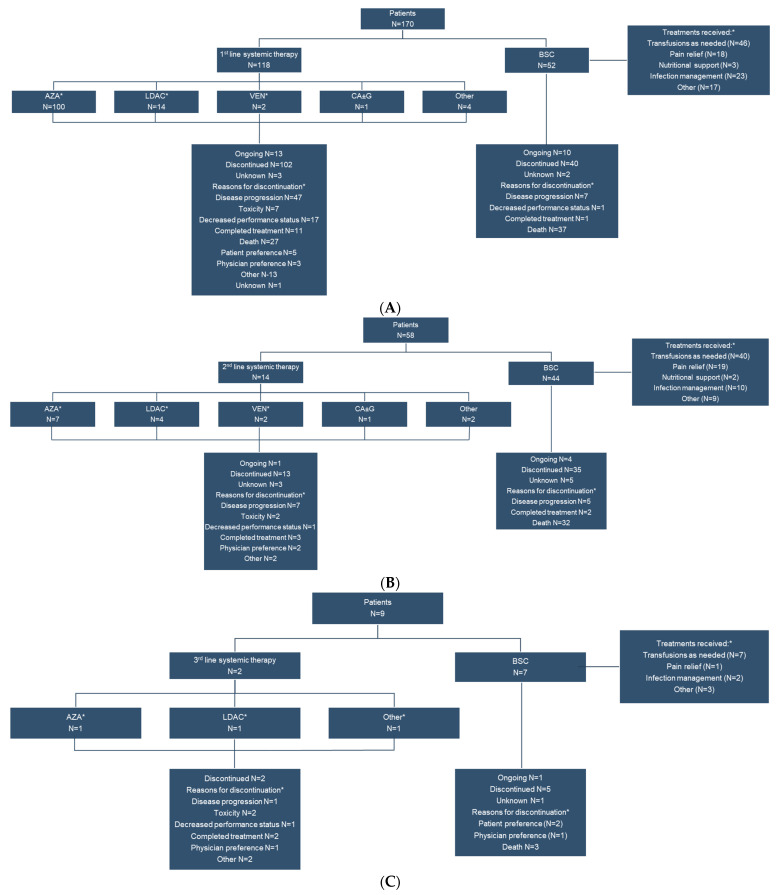
First line (**A**), second line (**B**) and third line (**C**) treatment patterns and disposition of Canadian patients ineligible for high intensity chemotherapy. * Patients may be taking more than one systemic therapy simultaneously. AZA, 5-azacitidine; BSC, best supportive care; CA ± G, cytarabine, aclarubicin, G-CSF regimen; G-CSF, Granulocyte—colony stimulating factor; LDAC, low-dose cytarabine; VEN, venetoclax.

The 118 patients who received systemic therapy as first line treatment received a median (range) of 5 (1–62) cycles, typically with AZA (n = 100, 84.7%) or LDAC (n = 14, 11.9%). This is markedly different from the distribution of patients receiving first-line systemic therapy in the global cohort (HMA: 61.8%, LDAC: 15.2%, Other [includes cytarabine, aclarubicin, G-CSF (CAG regimen), enocitabine, venetoclax, or combination therapies]: 23.1%). Fourteen patients received systemic therapy as second line treatment, but for a median (range) of 3.5 (1–27) cycles. Only two patients received a third line of systemic therapy (Figure 1). Among those who received BSC, the most common interventions were transfusions, other, infection management and pain relief (Figure 1). The most common reasons for discontinuation of systemic therapy were disease progression (n = 47, 46.1%), death (n = 27, 26.5%), decline in performance status (n = 17, 16.7%) and other (n = 13, 12.7%) for first line therapy. For second line systemic therapy the most common reasons for discontinuation were disease progression (n = 7, 53.8%) and completed planned treatment (n = 3, 23.1%). For those receiving BSC, the most common reason for treatment discontinuation was death (n = 37, 92.5% for first line, n = 32, 91.4% for second line treatment).

### 3.3. Overall Survival

**Figure 2:**
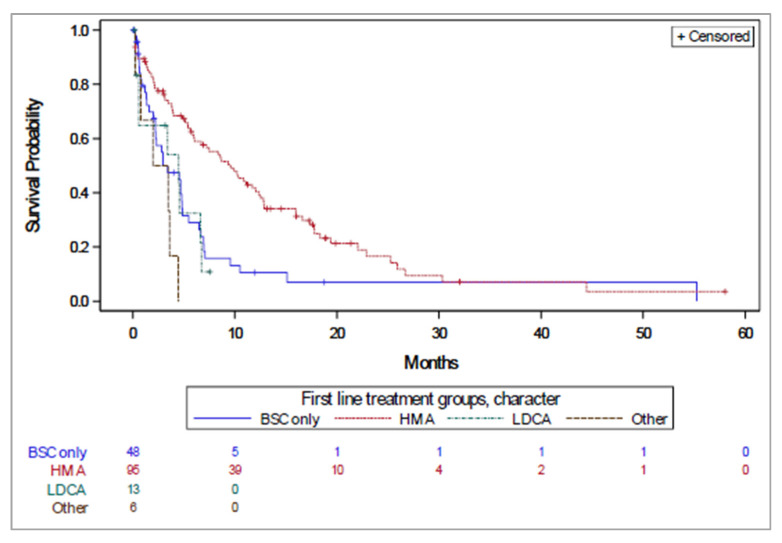
Time to treatment failure by first line treatment received.

The median OS for the overall population was 8.58 months (95% confidence interval [CI]: 6.2–11.1 months) and varied by first line treatment from 2.96 (2.2–4.9) months for BSC to 13.31 (10.0–15.2) months for HMAs (Figure 2, Table 2). For the global cohort, the longest median OS was also reported for the HMA group (9.9 months), followed by the LDAC group (7.9 months), other systemic therapy group (5.4 months), and finally the BSC group (2.5 months). Overall survival was 20.5% (95% CI: 13.8–28.4%) at two years and 3.2% (95 CI: 0.3–12.7%) at five years.

Overall, the most common cause of death was AML progression (68.9%), infection (17.2%) and unknown (8.2%), with similar rates for first-line systemic therapy and BSC (Table 3).

Similar survival patterns were observed for PFS and TTF (Table 2), with an apparent increase in median time to PFS and TTF for patients receiving first line HMAs, compared with those who received BSC, and LDAC intermediate.

### 3.4. Treatment Response

Best treatment response was unknown for approximately half of patients (Table 4). Few patients achieved a best overall response of CR or CRi with first or second line systemic therapy. It is interesting to note that two patients received venetoclax combination therapy as first line, and another two received it as second line, systemic therapy.

### 3.5. Healthcare Resource Utilization

During their first line of therapy, over 80% of patients had at least one outpatient visit with a median of 10 visits for patients receiving systemic therapy and 6 for those receiving BSC (Table 5). In addition, 79 (66.9%) patients receiving systemic therapy and 37 (71.2%) of those receiving BSC had at least one admission to hospital (the median number of hospitalizations per patient was 1 for both groups). The median duration of stay was 7 days for those receiving systemic therapy and 9 days for those receiving BSC. In the global cohort fewer patients had an outpatient visit with a similar median number of visits per patient (HMA: 79%; median: 13 visits, LDAC: 53%; median: 6 visits, Other: 63%; median: 11 visits, BSC: 66%; median: 6 visits). More patients had at least one admission to hospital and the median number of hospitalizations, and median duration, was higher (HMA: 82%; median: 6 visits, 8 days. LDAC: 93%; median: 5 visits, 16 days. Other: 83%; median: 4 visits, 18 days. BSC: 83%; median: 2 visits, 8 days). The most common reason for hospitalization during the first line of therapy in the Canadian dataset was infection-related (52.2% of admissions for patients receiving systemic therapy; 60.9% of those receiving BSC) while in the global cohort the most common reason for hospitalization was treatment administration for those receiving systemic therapy and infection-related for those receiving BSC. A total of 96 (81.4%) patients receiving first line systemic therapy and 39 (75.0%) of those receiving first line BSC had at least one RBC or platelet transfusion (median: 10 RBC transfusions for patients receiving systemic therapy and 6 for those receiving BSC. The median number of platelet transfusions was 1.5 for both groups). In general, these data align with those from the global cohort, although it is notable that the median number of platelet transfusions among patients receiving LDAC was 11.

Only 3 patients receiving their first line of systemic treatment had an assessment for MRD. All three assessments were performed on bone marrow aspirate samples.

Antibiotics or antivirals were used by approximately half of patients during their first line of therapy, almost always for curative purposes (Table 6). Use of antifungal therapy was much less common and occurred at least once in 21 (17.8%) and 3 (5.8%) of patients receiving first line systemic therapy and BSC, respectively. Anti-infective use was notably higher in the global CURRENT cohort: Antibiotics or antivirals were used by over 80% of patients receiving first line systemic therapy (HMA: 80%, LDAC: 92%, Other: 87%) and 72% of those receiving BSC, with higher use for prophylaxis (HMA:58%, LDAC, 49%, Other: 50%, BSC: 44%). Antifungals were used by a higher proportion of patients in the global cohort (HMA: 43%, LDAC: 63%, Other: 57%, BSC: 34%) with prophylaxis being the top reason for their use (HMA: 74%, LDAC: 67%, Other: 76%, BSC: 59%).

## 4. Discussion

This analysis of the Canadian dataset from the CURRENT non-interventional retrospective chart review highlights the real-world characteristics, treatment patterns, clinical outcomes and HRU of Canadians with AML who are unfit to receive intensive chemotherapy. As patients eligible for intensive chemotherapy were excluded from the study, this cohort provides an overview of the demographics and characteristics of patients on other therapeutic options. To mitigate possible sampling bias, specialist sites across Canada were approached to participate in the study and sites that identified more eligible patients than their enrolment target were provided with a random sampling method.

Approximately 40% were female, consistent with other Canadian data [22], and 60% were at least 75 years old at diagnosis. As would be expected for an older cohort, a high proportion (39%) had a poor cytogenetic risk profile [10], and just under 80% had at least one co-morbidity. Baseline characteristics for those patients who received first line systemic therapy were generally similar to those who elected BSC. The exception to this was performance status (which was worse for patients who received BSC), although it should be noted that performance status was unknown for 60% of patients who received systemic therapy compared with 29% of those who received BSC. Consistent with treatment recommendations when the medical records were generated, treatment options in this intensive chemotherapy-ineligible population were generally AZA, LDAC and BSC [6,7]. Among patients who received first line systemic therapy, use of HMAs was greater, and use of ‘Other’ therapies was lower, in the Canadian dataset than in the global cohort. This disparity appears to be related predominately to higher use of cytarabine, aclarubicin, granulocyte colony stimulating factor combination (CAG) outside of Canada (0.8% vs. 19%, respectively). Use of novel targeted agents was low in both cohorts, presumably reflecting the exclusion of patients who received treatment as part of a clinical trial and the limited availability of novel agents such as venetoclax or glasdegib for older patient during the study period. The proportion of Canadian patients who received a second line of systemic therapy was lower than that observed in the overall CURRENT cohort (12% vs. 18%, respectively). Among potential explanations for this is differences in reimbursement policies between participating countries (and also between Canadian Provincial drug plans).

Median survival was numerically longer in the Canadian cohort than in the overall CURRENT population (8.58 months vs. 6.2 months, respectively), which may reflect differences in demographics, baseline characteristics, use of systemic therapies and supportive care. Particularly intriguing is that in the Canadian dataset, more patients received HMAs and the OS was longer compared with the global cohort. This may reflect a treatment selection bias whereby in the global cohort patients with favourable disease and baseline characteristics received ‘Other’ treatment options instead of HMAs, such that the global HMA cohort may have contained patients with poorer disease and baseline characteristics. What was consistent was the abysmal life expectancy of patients selecting first line BSC, with a median OS of less than three months in both cohorts. Also consistent was the association between systemic therapy type and survival, with HMAs providing the longest OS. Overall, however, life expectancy and clinical outcomes in this population remain extremely poor compared with those who receive intensive therapy.

This analysis of healthcare resource utilization provided important data for non-intensively treated AML patients in Canada, and there are few other published multicenter Canadian studies in this area. The number of patients requiring at least one hospitalization was similar for first line systemic therapy and BSC, and although the proportion of patients requiring ≥3 hospitalizations was numerically higher for the systemic therapy group the median duration was longer, and there was a greater requirement for intensive care beds for those receiving BSC. This data suggests that use of a BSC strategy does not significantly reduce hospitalizations in Canada. As expected, there were more outpatient visits for patients receiving systemic therapy. This group required more transfusions, but this may reflect longer survival compared with patients who selected BSC. These data suggest real-world healthcare resource utilization may be only marginally impacted by treatment choice, unlike clinical outcomes. That being said, these inferences should be interpreted with caution as it is unknown what the impact on both survival and healthcare resource utilization would be if, for example, a significant proportion of patients who received BSC had received HMA therapy instead. It should also be noted that since these data were collected guidance has evolved such that BSC (hydroxyurea and transfusion support) is now only recommended as a final option for patients who are not candidates for intensive therapy and who have AML without actionable mutations [23]. Nevertheless, in clinical practice, it is necessary to consider patient preferences in decision making and occasionally patients will elect for BSC over other treatment options. The relatively low rates of anti-infective use, particularly in the context of prophylaxis, may reflect local institutional guidelines for anti-infective stewardship. In contrast to patients receiving intensive therapy or stem cell transplant, there is less robust evidence to support the use of prophylactic antimicrobials in the non-intensive setting and the low rate of use may reflect uncertainty to the benefit of this intervention.

The CURRENT study is one of the largest, global, real-world studies performed to date of treatment patterns in patients with AML who were ineligible for intensive chemotherapy. The Canadian dataset provides valuable insights into the real-world characteristics, treatment patterns, clinical outcomes and HRU of Canadians with AML who are unfit to receive intensive chemotherapy. Several limiting factors should be considered when interpreting these results. Real-world, retrospective studies are by nature observational, uncontrolled and nonrandomized, and missing data limit the implications of some endpoints. Molecular and cytogenetic data, and performance status, were often not recorded, which limited assessment of their impact on outcomes, and some endpoints (e.g., type of BSC provided, best overall response achieved) elicited a high number of responses as “other” or “unknown” which challenged interpretation. While the six Canadian sites provide strong regional representation, there was no site from Atlantic Canada. All the sites involved in the study were academic centers and as such may not necessarily represent treatment patterns or outcomes for patients treated in rural areas and smaller centers. Within participating sites there was also the potential for selection bias when considering patients’ medical records for data extraction. Sample size considerations obviated the potential for exploration of regional differences. Finally, the data capture period for the study preceded approval of newer targeted therapies in many jurisdictions and thereby provided an assessment of treatments that may now be considered foundational. Indeed, the advent of novel therapies may enable more patients considered unfit for existing intensive treatment options to still achieve remission thereby allowing them to be considered as candidates for transplant.

## 5. Conclusions

Overall, this analysis confirms that historical outcomes in patients with AML who were ineligible for intensive chemotherapy were poor, with HMAs demonstrating a benefit over alternatives. As the incidence of AML rises consequent to an aging population, so does the number of patients who are ineligible for intensive chemotherapy, highlighting the clinical need for novel agents and combination therapies that are both effective and appropriate for use in this treatment-challenged population.

## Figures and Tables

**Table 1:** Patient demographics and baseline characteristics.

	Overall (n = 170)	1st Line Systemic Therapy (n = 118)	BSC (n = 52)
Female gender (n [%]) *	65 (38.2)	41 (34.7)	24 (46.2)
Mean (SD) age at diagnosis (years)	74.3 (7.01)	74.3 (6.90)	74.3 (7.37)
≤75 years (n [%])	99 (58.2)	71 (60.2)	28 (53.8)
Secondary AML			
Yes	73 (42.9)	44 (37.3)	29 (55.8)
No	79 (46.5)	59 (50.0)	20 (38.5)
Unknown	18 (10.6)	15 (12.7)	3 (5.8)
ECOG performance status			
0–1	41 (24.1)	27 (22.9)	14 (26.9)
≥2	43 (23.5)	20 (17.0)	23 (44.2)
Unknown	86 (50.6)	71 (60.2)	15 (28.8)
AML classification—WHO (n [%])			
AML with recurrent abnormalities	13 (7.6)	11 (9.3)	2 (3.8)
AML with MDS-related changes	76 (44.7)	53 (44.9)	23 (44.2)
AML not otherwise specified	41 (24.1)	28 (23.7)	13 (25.0)
Myeloid sarcoma	1 (1.2)	0	2 (3.8)
Unknown	33 (19.4)	22 (18.6)	11 (21.2)
Cytogenetic risk (n [%])			
Favourable	26 (15.3)	19 (16.1)	7 (13.5)
Intermediate	47 (27.6)	38 (32.2)	9 (17.3)
Poor	61 (35.9)	46 (39.0)	15 (28.8)
Unknown	36 (21.2)	15 (12.7)	21 (40.4)
Molecular features identified (n [%])			
Any	40 (23.5)	30 (25.4)	10 (19.2)
IDH2	1 (2.5)	1 (3.3)	0
TP53	2 (5.0)	2 (6.7)	0
TET2	1 (2.5)	1 (3.3)	0
RUNX1	5 (12.5)	3 (10.0)	2 (20.0)
DNMT3A	1 (2.5)	1 (3.3)	0
ASXL 1	3 (7.5)	3 (10.0)	0
FLT3^TKD^	2 (5.0)	2 (6.7)	0
JAK2	7 (17.5)	3 (10.0)	4 (40.0)
NPM1	13 (32.5)	10 (33.3)	3 (30.0)
SRSF2	2 (5.0)	2 (6.7)	0
MLLPTD	2 (5.0)	1 (3.3)	1 (10.0)
Other	11 (27.5)	9 (30.0)	2 (20.0)
None	56 (32,9)	42 (35.6)	14 (26.9)
Unknown	74 (43.5)	46 (39.0)	28 (53.8)
Co-morbidities (n [%])			
Myocardial infarction	2 (1.2)	1 (0.8)	1 (1.9)
Angina/coronary artery disease	22 (12.9)	12 (10.2)	10 (19.2)
Congestive heart failure	11 (6.5)	8 (6.8)	3 (5.8)
Arrhythmias	14 (8.2)	9 (7.6)	5 (9.6)
Restrictive lung disease or COPD	8 (4.7)	8 (6.8)	0
Liver cirrhosis (Child Pugh A, B or C)	1 (0.6)	1 (0.8)	0
Elevated transaminases unrelated to cirrhosis	1 (0.6)	1 (0.8)	0
CKD stage 3, 4 or 5	2 (1.2)	0	2 (3.8)
Other	99 (58.2)	69 (58.5)	30 (57.7)
Unknown	15 (8.8)	9 (7.6)	6 (11.5)
None	39 (22.9)	27 (22.9)	12 (23.1)

* All patients in the Canadian dataset identified as either male or female. AML, acute myeloid leukemia; ASXL 1, ASXL transcriptional regulator 1; BSC, best supportive care; CKD, chronic kidney disease; COPD, chronic obstructive pulmonary disease; DNMT3A, DNA methyltransferase 3 alpha; FLT3^TKD^, FLT3 tyrosine kinase domain; IDH2, isocitrate dehydrogenase 2; JAK2, Janus kinase 2; MDS, myelodysplastic syndrome; MLLPTD, mixed-lineage leukemia gene-partial tandem duplication; NPM1, nucleophosmin 1; RUNX1, runt-related transcription factor 1; SRSF2, serine and arginine rich splicing factor 2; TET2, tet methylcytosine dioxygenase 2; TP53, tumor protein P53; WHO, whorl health organization.

**Table 2:** Kaplan–Meier estimates of survival.

	Overall (n = 170)	LDAC (n = 14)	HMA (n = 97)	Other (n = 7)	BSC Only (n = 52)
Median (95% CI) OS (months)	8.6 (6.2–11.1)	6.4 (5.0–14.2)	13.1 (10.0–15.2)	NE	3.0 (2.2–4.9)
Median (95% CI) PFS (months)	5.8 (4.4–7.2)	5.5 (1.4–12.9)	9.7 (7.2–11.4)	3.6 (1.5–NE)	2.4 (1.2–3.2)
2-year (95% CI) OS (%)	20.5 (13.8–28.2)	11.7 (0.7–39.4)	26.9 (17.2–37.4)	62.5 (14.2–89.3)	6.7 (1.4–17.9)
5-year (95% CI) OS (%) *	3.2 (0.3–12.7)	0	4.7 (0.5–17.4)	0	0

* Last observation was censored before Month 60; results for Month 59 are presented here. BSC, best supportive care; HMA, hypomethylating agents; LDAC, low-dose cytarabine; OS, overall survival; PFS, progression-free survival.

**Table 3:** Patient outcomes at the end of study.

	Overall (n = 170)	1st Line Systemic Therapy (n = 118)	BSC Only (n = 52)
Alive at end of study	48 (28.2)	37 (31.4)	11 (21.2)
Cause of death (n [%]):			
AML progression	84 (68.9)	56 (69.1)	28 (68.3)
Infection	21 (17.2)	16 (19.8)	5 (12.2)
Multi-organ failure	1 (0.8)	16 (19.8)	1 (2.4)
Other comorbid conditions	5 (0.1)	3 (3.7)	2 (4.9)
Unrelated to a disease	1 (0.8)	3 (3.7)	1 (2.4)
Unknown	10 (8.2)	6 (7.4)	4 (9.8)

AML, acute myeloid leukemia; BSC, best supportive care.

**Table 4:** Best overall response to first and second line therapy.

Best Overall Response (n, %)	First Line Therapy (n = 118)	Second Line Therapy (n = 14)
CR	10 (8.5)	1 (7.1)
Cri	8 (6.8)	0
PR	18 (15.3)	4 (28.6)
SD	21 (17.8)	0
PD	12 (10.2)	2 (14.3)
Unknown	49 (41.5)	7 (50.0)

CR, complete remission; Cri, CR with incomplete hematologic recovery; PD, progressive disease; PR, partial remission; SD, stable disease.

**Table 5:** Healthcare resource utilization during first line therapy.

	First Line Systemic Therapy (n = 118)	BSC (n = 52)
Outpatient consultation (N [%]):		
Yes *	96 (81.4)	43 (82.7)
No	18 (15.3)	5 (9.6)
Unknown	4 (3.4)	4 (7.7)
Number of visits (median [range])	10 (1–105)	6 (1–80)
Hospitalization (N [%]):		
Yes *	79 (66.9)	37 (71.2)
No	36 (30.5)	8 (15.4)
Unknown	3 (2.5)	7 (13.5)
Number of hospitalizations (N [%]):		
1	50 (63.3)	27 (73.0)
2	19 (24.1)	7 (18.9)
≥3	10 (12.7)	3 (8.1)
Duration of stay (days, median [range])		
Overall	7 (1–100)	9 (1–92)
In ICU	0 (0–31)	2 (0–38)
Reason for hospitalization: ^†^		
Progression/relapse-related	22 (16.4)	19 (34.5)
Infection-related	70 (52.2)	32 (58.2)
Transfusion-related	9 (6.7)	4 (7.3)
Treatment administration-related	14 (10.4)	3 (5.5)
Other AML-related	25 (18.7)	10 (18.2)
Other	38 (28.4)	10 (18.2)
RBC/PLT transfusion (N [%]):		
Yes	96 (81.4)	39 (75.0)
No	16 (13.6)	7 (13.5)
Unknown	6 (5.1)	6 (11.5)
If yes, number of RBC transfusions (median [range])	10 (2–180)	6 (1–100)
If yes, number of PLT transfusions (median [range])	1.5 (0–50)	1.5 (0–200)

* Where applicable, this value is used as the denominator for calculating percentages, and only those patients with at least one outpatient consultation/hospitalization were included in calculations of medians and ranges for number of visits and length of stay, respectively. ^†^ Multiple selections were possible. AML, acute myeloid leukemia; BSC, best supportive care; ICU, intensive care unit; PLT, platelet; RBC, red blood corpuscle.

**Table 6:** Anti-infective use.

	Systemic Therapy	BSC
**First Line Therapy**	**n = 118**	**n = 52**
Antibiotic or antiviral use (N [%]):		
Yes *	63 (53.4)	29 (55.8)
No	54 (45.8)	16 (30.8)
Unknown	1 (0.8)	7 (13.5)
Reason for use:^†^		
Prophylaxis	16 (25.4)	3 (10.3)
Curative	51 (81.0)	27 (93.1)
Unknown	3 (4.8)	0
Antifungal use (N [%]):		
Yes*	21 (17.8)	3 (5.8)
No	96 (81.4)	44 (84.6)
Unknown	1 (0.8)	5 (9.6)
Reason for use:^†^		
Prophylaxis	11 (52.5)	1 (33.3)
Curative	8 (38.1)	2 (66.7)
Unknown	2 (9.5)	0
**Second Line Therapy**	**n = 14**	**n = 44**
Antibiotic or antiviral use (N [%]):		
Yes*	6 (42.9)	17 (38.6)
No	7 (50.0)	21 (47.7)
Unknown	1 (7.1)	6 (13.6)
Reason for use:^†^		
Prophylaxis	1 (16.7)	6 (35.3)
Curative	6 (100.0)	10 (58.8)
Unknown	0	2 (11.8)
Antifungal use (N [%]):		
Yes *	2 (14.3)	8 (18.2)
No	12 (85.7)	30 (68.2)
Unknown	0	6 (13.6)
Reason for use:^†^		
Prophylaxis	1 (50.0)	4 (40.0)
Curative	1 (50.0)	3 (37.5)
Unknown	0	1 (12.5)
**Third Line Therapy**	**n = 2**	**n = 7**
Antibiotic or antiviral use (N [%]):		
Yes *	2 (100.0)	3 (42.9)
No	0	4 (57.1)
Reason for use: ^†^		
Prophylaxis	1 (50.0)	0
Curative	1 (50.0)	3 (100.0)
Unknown	1 (50.0)	0
Antifungal use (N [%]):		
Yes *	2 (100.0)	2 (28.6)
No		5 (71.4)
Reason for use: ^†^		
Prophylaxis	1 (50.0)	0
Curative	1 (50.0)	2 (100.0)
Unknown	1 (50.0)	0

* Value used as the denominator for calculating percentages. ^†^ Multiple selections were possible. BSC, best supportive care.

## Data Availability

AbbVie is committed to responsible data sharing regarding the clinical trials we sponsor. This includes access to anonymized, individual, and trial-level data (analysis data sets), as well as other information (e.g., protocols and Clinical Study Reports), as long as the trials are not part of an ongoing or planned regulatory submission. This includes requests for clinical trial data for unlicensed products and indications. This clinical trial data can be requested by any qualified researchers who engage in rigorous, independent scientific research, and will be provided following review and approval of a research proposal and Statistical Analysis Plan (SAP) and execution of a Data Sharing Agreement (DSA). Data requests can be submitted at any time and the data will be accessible for 12 months, with possible extensions considered. For more information on the process, or to submit a request, visit the following link: https://www.abbvie.com/our-science/clinical-trials/clinical-trials-data-and-information-sharing/data-and-information-sharing-with-qualified-researchers.html, accessed on 4 April 2022.
